# Interaction between decision-making and motor learning when selecting reach targets in the presence of bias and noise

**DOI:** 10.1371/journal.pcbi.1011596

**Published:** 2023-11-02

**Authors:** Tianyao Zhu, Jason P. Gallivan, Daniel M. Wolpert, J. Randall Flanagan

**Affiliations:** 1 Centre for Neuroscience Studies, Queen’s University, Kingston, Ontario, Canada; 2 Department of Psychology, Queen’s University, Kingston, Ontario, Canada; 3 Department of Biomedical and Molecular Sciences, Queen’s University, Kingston, Ontario, Canada; 4 Zuckerman Mind Brain Behavior Institute, Columbia University, New York, New York; 5 Department of Neuroscience, Columbia University, New York, New York; Chinese Academy of Sciences, CHINA

## Abstract

Motor errors can have both bias and noise components. Bias can be compensated for by adaptation and, in tasks in which the magnitude of noise varies across the environment, noise can be reduced by identifying and then acting in less noisy regions of the environment. Here we examine how these two processes interact when participants reach under a combination of an externally imposed visuomotor bias and noise. In a center-out reaching task, participants experienced noise (zero-mean random visuomotor rotations) that was target-direction dependent with a standard deviation that increased linearly from a least-noisy direction. They also experienced a constant bias, a visuomotor rotation that varied (across groups) from 0 to 40 degrees. Critically, on each trial, participants could select one of three targets to reach to, thereby allowing them to potentially select targets close to the least-noisy direction. The group who experienced no bias (0 degrees) quickly learned to select targets close to the least-noisy direction. However, groups who experienced a bias often failed to identify the least-noisy direction, even though they did partially adapt to the bias. When noise was introduced after participants experienced and adapted to a 40 degrees bias (without noise) in all directions, they exhibited an improved ability to find the least-noisy direction. We developed two models—one for reach adaptation and one for target selection—that could explain participants’ adaptation and target-selection behavior. Our data and simulations indicate that there is a trade-off between adaptation and selection. Specifically, because bias learning is local, participants can improve performance, through adaptation, by always selecting targets that are closest to a chosen direction. However, this comes at the expense of improving performance, through selection, by reaching toward targets in different directions to find the least-noisy direction.

## Author summary

In the real world, movement errors can result from both bias (systematic error) and noise (random error). A fundamental challenge for the sensorimotor system is to make appropriate changes in behavior when facing these two sources of errors. To examine this issue, we developed a center-out reaching task in which participants could select one of three randomly positioned targets. An angular displacement was added between viewed and actual reach endpoint position. The displacement included a constant bias equal in all directions and a noise of which the amplitude depended on the selected target direction. For different participant groups, we applied different bias angles (0 to 40 degrees). Participants who experienced larger bias angles were less successful at finding the least-noisy target direction. In a second experiment, when participants adapted to a 40 degrees bias in all directions before experiencing target-dependent noise, they were more successful at finding the least-noisy direction than participants—in the first experiment—who experienced bias and noise simultaneously. Our results indicate that participants cannot disentangle the contribution of bias and noise to their movement errors, such that adaptation to bias limits their ability to select less-noisy movements.

## Introduction

Skilled motor performance requires the ability to adapt to perturbations imposed by the environment. These perturbations can be deterministic (e.g. a bias) or stochastic (e.g. noise). Computationally, adaptation to deterministic perturbations involves error-based learning, driven by signed error signals, to compensate for the bias. Examples of deterministic perturbations include visuomotor rotations applied to the viewed position of the hand [1–3] and force-fields applied to the hand or arm [4–6]. In contrast, adaptation to stochastic perturbations involves reinforcement-based learning, driven by reward or loss, to either compensate for or reduce noise. For example, when pointing to a screen that includes reward and penalty regions, people can learn to compensate for the current motor noise level so as to select an aim point that maximizes expected reward [7–9]. When different levels of noise are associated with different alternative movements, people can learn to select the movement that minimizes the noise and leads to the best expected outcome [10–14]. Note that unlike the reduction of bias, where the bias itself indicates the direction of correction for the movement, the reduction of noise is driven by the unsigned loss signal (i.e., the amplitude of noise) and necessarily involves exploration of different movement possibilities.

A few studies have examined motor learning under both bias and noise, focusing on how noise affects bias adaptation. When noise increases the uncertainty of the feedback signal within a single trial (e.g., increased blurriness of the visual feedback), adaptation rate has been observed to decrease [15, 16], as predicted by Bayesian integration models [17, 18]. Adaptation rate can also decrease when noise reduces across-trial consistency (e.g., perturbation varying randomly across trials with a constant mean) [19]. Moreover, asymptotic or residual error between adaptation and bias is larger when the noise is larger [20].

There are situations, however, when both bias and noise need to be reduced. Consider playing pétanque (a French throwing game in which metal balls are thrown to get as near as possible to a small wooden ball called the cochonnet) on an uneven surface. In this scenario, the total loss (ball to cochonnet distance) is a combination of the inaccuracy of the shot (i.e. bias in the location the ball lands relative to the desired location) and the variability (i.e. noise) after the ball lands resulting from the unevenness of the playing surface, which can vary with location. To be optimal when throwing to a particular cochonnet location, a player would need to take into account how both their throwing bias and the surface-induced variability vary across locations. This would allow them to choose an optimal desired location trading off bias and variance. Therefore, to be optimal requires exploration of different desired locations so as to reduce bias—because bias learning is typically local—and to learn the variability structure of the environment.

**Fig 1:**
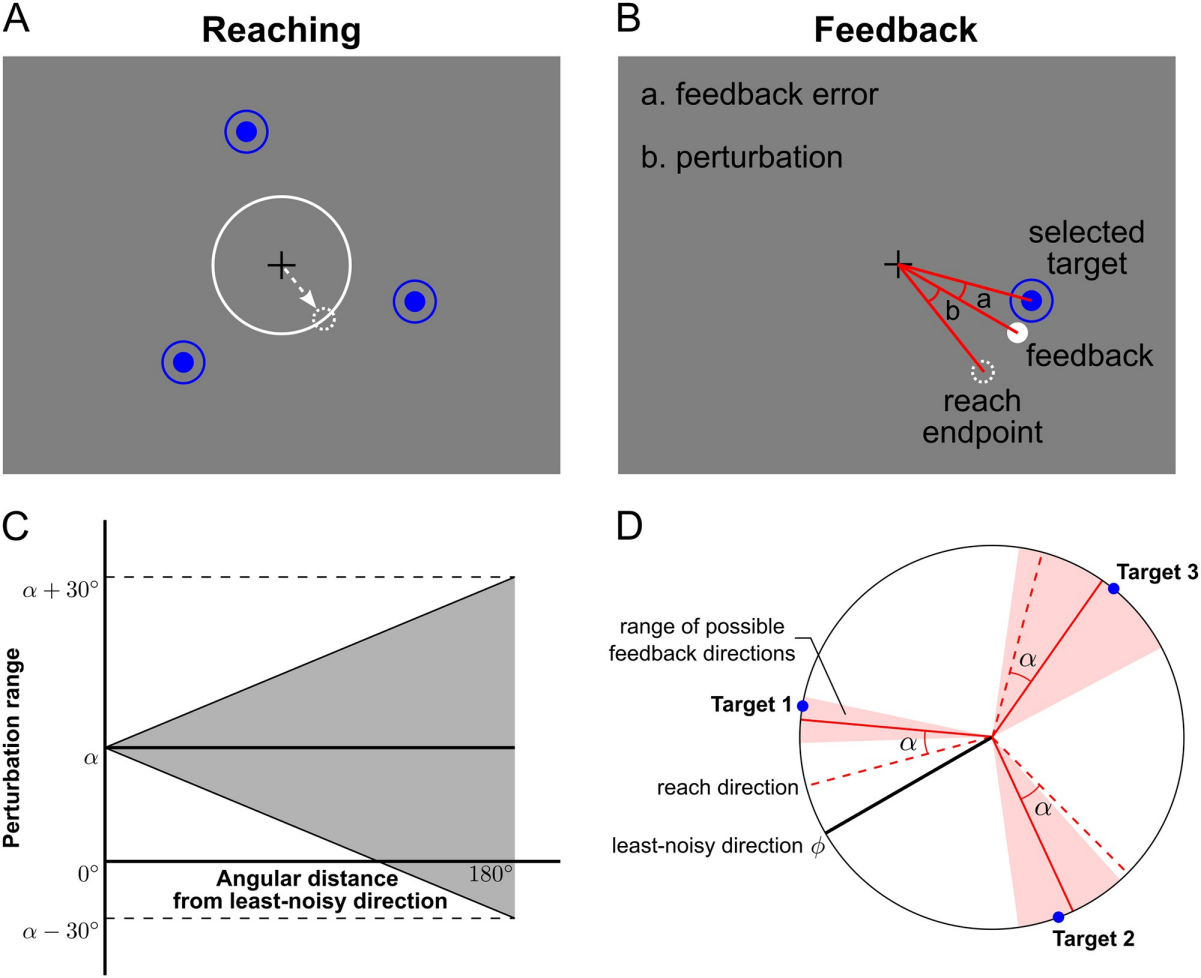
Behavioral paradigm. A: Illustration of a choice trial. The participant was asked to make a center-out reaching movement (dashed white arrow) toward one of three potential targets (blue circles with central dot). During reaching, the cursor position was hidden (dashed white circle shows hidden location) and a white expanding circle indicated the distance (but not direction) of the cursor from the central location (cross). B: At the end of the movement, visual feedback of the static cursor position was displayed. The cursor position was displayed offset from the actual position (dashed circle, not shown to participants) by an angular perturbation. C: Perturbation range for targets with different absolute angular distances from the least-noisy direction (*ϕ*). The perturbation was composed of a rotation offset *α* (constant in all directions, here shown for *α* = 20°) and a target-direction-dependent noise. The noise was drawn from a uniform distribution with a range that linearly increased with the angular distance of the selected target from direction *ϕ*. D: Possible feedback range (pink) given the selected target (blue dots) and the reach direction (dashed red lines). For each participant, a direction *ϕ* (solid black line) was randomly selected and was fixed throughout the experiment. The farther the selected target is from this direction, the greater the variability of the cursor perturbation.

The goal of our study was to examine how people adapt their movements when facing both bias and noise. To this end, we examined how participants chose a desired target direction in a center-out reaching task in which we included constant bias for all targets (akin to starting with the same bias for each landing location in pétanque) and target-direction-dependent noise (akin to a surface where the unevenness varies with location). Fig 1 illustrates the behavioral paradigm. A visuomotor rotation was imposed on each trial which had both a constant (bias) component and a variable (noise) component, with the latter depending on the target direction. Crucially, on each trial participants could select one of three randomly positioned reach targets. This allowed us to assess both the learning of the constant rotation (for different target directions) and whether participants could estimate the noise structure so as to choose the target in the least-noisy direction. In particular we examined whether bias learning affected estimation of the noise structure. Note that we recognize that in real-world tasks, both bias and noise can vary to different degrees across movement options. In our paradigm we applied constant bias (with a magnitude that varies across groups) and noise that varies across movement options, a scenario that approximates a subset of real-world tasks.

**Fig 2:**
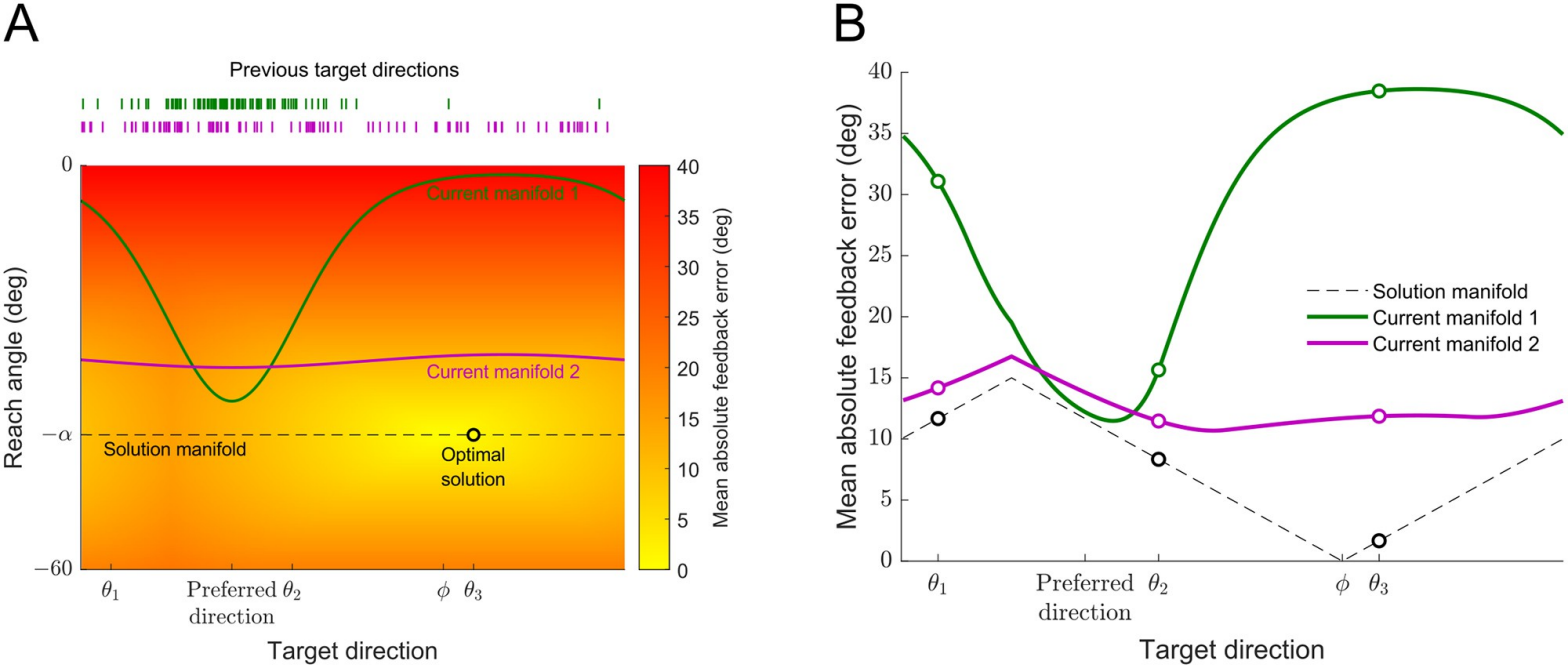
The interplay of learning the bias angle and learning the least-noisy direction. A: Mean absolute feedback error (red-yellow gradient) as a function of target direction (abscissa) and the reach angle relative to the target (ordinate) for a visuomotor bias angle *α* and a least-noisy direction *ϕ*. The dashed black line shows the solution manifold for which the expected signed error is 0, that is the reach angle (−*α*) compensates for the bias angle. Absolute error increases away from the least-noisy direction (*ϕ*) and away from the solution manifold, with the black circle marking the optimal solution given the optional target directions in the trial (*θ*_1_, *θ*_2_ and *θ*_3_). Green and purple curves show example manifolds (reach angle as a function of target direction) for two notional participants who have selected different distributions of target choices (each tick in the top two rows denoting a selected target direction). B: Corresponding mean absolute feedback error as a function of selected target direction for the solution manifold and for the two notional participants shown in Panel A.

Fig 2A illustrates how two notional participants might perform in the task. In our experiment participants chose a target direction and a reach angle for that target. The mean absolute feedback error (shown in red-yellow gradient) depends on i) the target direction (which determines variability) and ii) the reach angle (which determines the remaining bias). When the visuomotor bias is *α*, choosing a reach angle (reach direction relative to target direction) of −*α* leads to a mean *signed* error of 0 for any target. We call the set of all target directions and reach angles that lead, on average, to no signed error the *solution manifold* (black dashed line). For an example trial in which the three optional targets are located in directions *θ*_1_, *θ*_2_ and *θ*_3_, the optimal solution (black circle) that will lead to smallest mean *absolute* error is to select the target closest to the least-noisy direction *ϕ* (the target in *θ*_3_) together with a reach angle of −*α*.

If participants can completely decompose the bias and noise components of the feedback error signal, they will be able to learn the solution manifold via error-based learning driven by the bias signal, and learn the optimal solution via reinforcement learning driven by the noise amplitude signal. However, participants are not likely to be able to learn the whole solution manifold within a limited number of trials. Consider two notional participants midway through an experiment who have chosen different distributions of target directions over their previous trials (shown in Fig 2A top). The green and purple curves in the parameter space show the current relationship between the reach angle and the target direction for these two participants (which we call *current manifolds*). The green curve is for a participant who has extensively practiced in a “preferred direction” far from the least-noisy direction (*ϕ*) leading to the reach angle compensating for the bias locally in this preferred direction. In contrast the purple participant has practiced more uniformly leading to them compensating for the bias for all target directions (although less compensation than for the green participant at their preferred direction). Fig 2B plots the corresponding mean absolute feedback error along the solution manifold (black dashed line) as well as along the two notional participants’ current manifolds as a function of the target direction. On the solution manifold the mean absolute feedback error is equal to the noise amplitude. For the two notional participants, the mean absolute feedback error is a combination of the noise amplitude and the remaining bias (depending on the current reach angle) in each target direction. The green notional participant is likely to choose targets close to the preferred direction (e.g., the target in *θ*_2_) since the mean absolute feedback error is smallest for that target. This may discourage them from exploring other target directions, since errors will be large there, preventing them from learning the bias for other target directions and finding the truly optimal solution. Therefore, this participant may be stuck in a local minimum. In contrast, the purple participant has a gentler gradient of error (e.g., the targets in *θ*_2_ and *θ*_3_ lead to very similar mean absolute feedback errors), allowing them to explore more and find the optimal solution.

We performed two web-based experiments based on this paradigm in which participants completed the task using a mouse or trackpad. In Experiment 1, we applied different bias angles (0°–40°) to different groups of participants (but the same target-direction-dependent noise). This allowed us to assess how bias and noise learning interact. Without bias, many participants found the optimal (least-noisy) direction. However, as the bias increased, participants tended to choose sub-optimal targets away from the least-noisy direction, indicating an interaction between bias and noise learning. To examine whether learning the bias alone prior to choosing targets improves target selection, in Experiment 2, we trained a group of participants first to adapt to the bias in all target directions before letting them freely select targets. We found that pre-training allowed this group to learn the optimal solution better than the corresponding group from Experiment 1. We developed two interacting models—of reach adaptation and target selection—which can account for our participants’ behavior.

## Results

### Experiment 1: Learning in the presence of visuomotor rotation bias and noise

In Experiment 1, participants were presented with three target options—equidistant from the central start position—and were required to select a target and move the cursor to it. The target locations were randomized but the spacing between them was always 120°. During the reach, the cursor was extinguished and only displayed at the end of the movement (Fig 1A). On each trial, an angular perturbation was introduced between the hand position and the displayed cursor position (Fig 1B). This perturbation included both a bias component—a constant visuomotor rotation angle—and a noise component—a randomly selected rotation angle from a uniform distribution. Crucially, the noise was target-direction dependent, with a direction *ϕ* where there was no noise and with the noise increasing with the angular distance from *ϕ* (Fig 1C). Therefore, participants could reduce the noise component by selecting the target closest to the least-noisy direction *ϕ* (Fig 1D). Across five groups of participants we varied the bias of the perturbation (0°, 10°, 20°, 30° and 40°).

**Fig 3:**
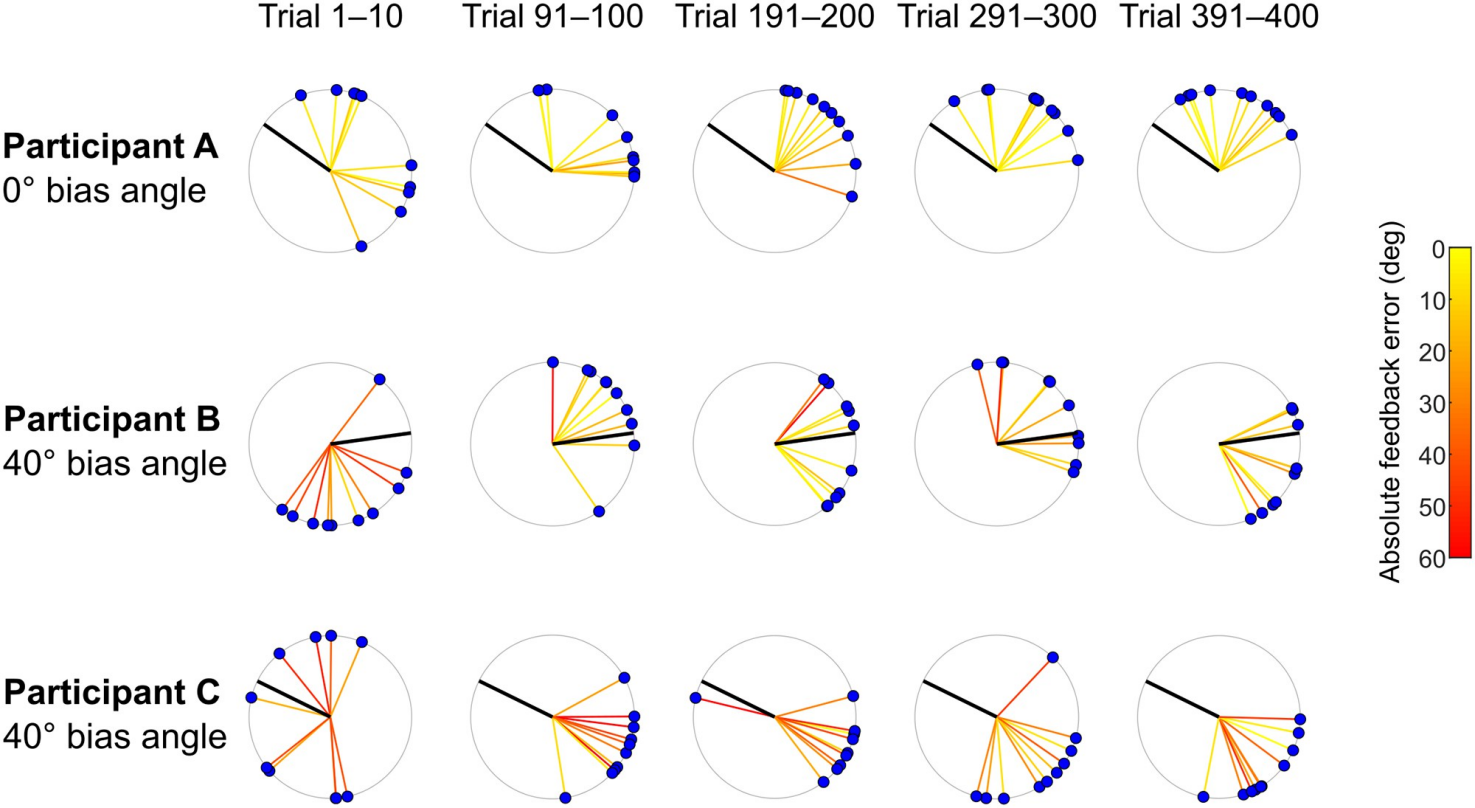
Examples of participants’ task performance. Target choices on individual trials were shown by the blue dots and color of the yellow-to-red lines associated with each dot represents the feedback error amplitude. Participant A performed at an average level in learning the least-noisy direction (direction *ϕ*, thick black line) among participants who experienced 0° bias. Participants B and C performed the best and worst in learning direction *ϕ* among participants who experienced the 40° bias.

Fig 3 shows examples of three participants’ task performance in different phases of the experiment. The blue dots represent the selected target, the black line represents direction *ϕ*, and the colored lines represent feedback error. Among participants who experienced the 0° bias, participant A performed at an average level in terms of learning to select targets close to direction *ϕ*. This participant showed modest learning across phases in that the selected targets got closer to *ϕ*. Among participants who experienced the 40° bias, participants B and C were the best and worst in terms of learning to select targets close to *ϕ*. The target choices of participant B gradually became quite close to direction *ϕ* whereas the target choices of participant C became concentrated in a direction opposite to direction *ϕ* by the end of the experiment. Note that the feedback errors were initially large for the two participants who experienced the 40° bias.

**Fig 4:**
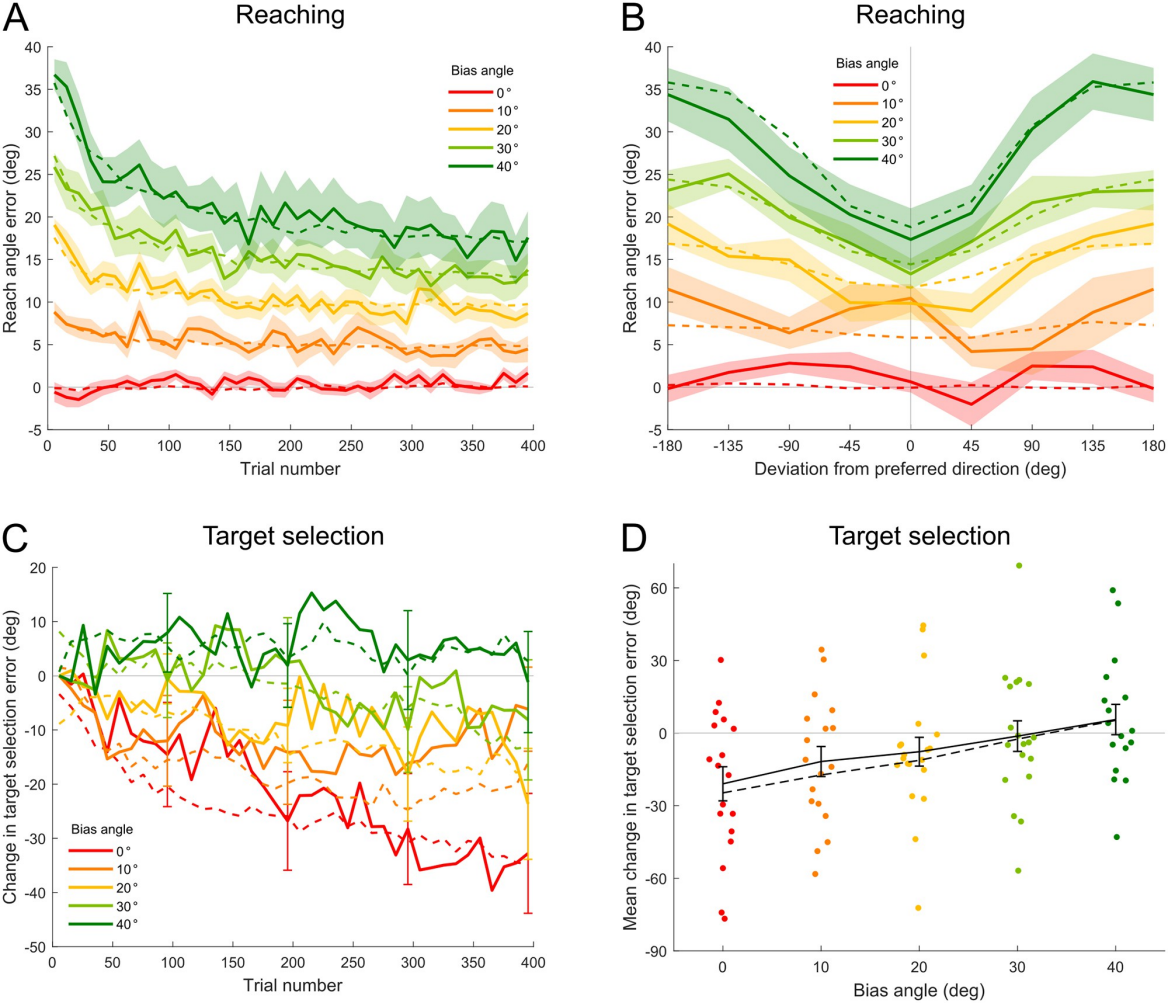
Participants’ reaching and target selection behavior across bias angles. A: Reach angle error as a function of trial number (in consecutive 10-trial bins). Solid curves show empirical results (mean ± *SEM* across participants) and dashed curves show model fits. B: Reach angle error as a function of the deviation from the participants’ preferred direction for probe trials. Solid curves show empirical results (45° bins, mean ± *SEM* across participants) and dashed curves show model fits. C: Change in target selection error as a function of trial number (in consecutive 10-trial bins). Solid curves show empirical results (mean ± *SEM*; for clarity *SEM* only shown at the end of each block) and dashed curves show model fits. D: Mean change in target selection error across the experiment. Colored dots show individual participant’s data. Black solid curve shows mean ± *SEM* across participants. Black dashed curve shows mean of model fits.

We first examined participants’ reaching behavior under different bias angles. We quantified reaching performance by the *reach angle error*, the angular deviation between the actual hand endpoint and the ideal hand endpoint that would compensate fully for the bias (i.e. would on average be most accurate given the noise). Fig 4A shows the time course of the reach angle error for each group. When the bias angle was non-zero, there was clear visuomotor adaptation, although adaptation was incomplete within the 400 trials. Moreover, reach angle error appears to level off at an asymptote that increases with the bias angle (correlation between bias angle and mean reach angle error across last 50 trials for each participant: Pearson’s *r* = 0.64, *p* = 10^−11^). Note that the dashed curves represent model fits which will be described below.

To assess reaching performance for different directions, we examined the 24 probe trials at the end of the experiment. On probe trials (3 per direction), participants reached toward a single target in one of 8 test directions and endpoint feedback was not provided. Fig 4B shows the reach angle error as a function of the angular deviation of the reach target from each participant’s preferred direction (circular mean of the last 50 target choices in the experiment). For the larger bias angles (20°, 30° and 40°), the reach angle error was lowest near the preferred direction and increased with the angular distance from that direction. This suggests that the learning did not generalize to directions where the participant rarely practiced. To test the effect of bias angle on the learning outcomes in different directions, we constructed a mixed-effect linear model fitting the reach angle error as a function of the absolute angular distance from the test direction to the preferred direction, the bias angle, and their interaction. The model included participant-specific random intercepts and random slopes for the angular distance to the preferred direction. The coefficient for the bias angle is significant (*p* = 10^−7^), showing that the reach angle error increased with the bias angle. The interaction between the bias angle and the absolute angular distance from the test direction to the preferred direction is also significant (*p* = 10^−8^), showing that as the test direction got farther from the preferred direction, the reach angle error increased faster when the bias angle was larger.

We then examined participants’ target selection behavior. We compared participants’ performance in learning the least-noisy direction *ϕ* across different visuomotor rotation bias angles. To evaluate the learning process, we defined the *target selection error* in each trial as the absolute angular distance between *ϕ* and the target selected by the participant, subtracting the absolute angular distance between *ϕ* and the least-noisy target (the target closest to direction *ϕ*) in that trial. Therefore the target selection error is zero if the target closest to *ϕ* is chosen, and if another target is selected it is a measure of how much worse that target is relative to the best target they could have chosen. Note that if *ϕ* is approximately midway between two targets, such that the two targets are similarly far away, the error linked with selecting the second closest target will be small. The range of the target selection error is 0°–120°.

For each participant, we calculated the mean target selection error in each consecutive 10-trial bin in the experiment. However, the initial distribution of target choices (as well as the corresponding distance measurement) varied widely across participants (see Fig 3 leftmost column). To correct for this initial target choice difference, we subtracted the mean target selection error in the first bin of each participant from all bins in the experiment for that participant. That is, we focused on the change in the target selection error. A negative value means that the current target choices are closer to direction *ϕ* relative to the initial target choices and implies learning of direction *ϕ*.

Fig 4C shows the change in target selection error as a function of trial number. When the bias angle was 0°, there was clear learning of direction *ϕ* on the group level. As the bias angle increased, the learning of direction *ϕ* became weaker. To quantify learning, we calculated the mean change in target selection error across all bins in the experiment for each participant. This measurement takes into account both the rate and amplitude of learning direction *ϕ*. Fig 4D shows this mean change for each participant in different groups. The mean change in target selection error became significantly more positive as bias angle increased (Pearson’s *r* = 0.32, *p* = 0.0022), showing that the learning of direction *ϕ* was inhibited by large bias angles.

### Modeling the learning process

We developed a model to account for participants’ behavioral results. This model consists of two independent processes—one determines the reach angle given a selected target (reach model), while the other determines which target to select (target selection model).

#### Model for reaching

**Fig 5:**
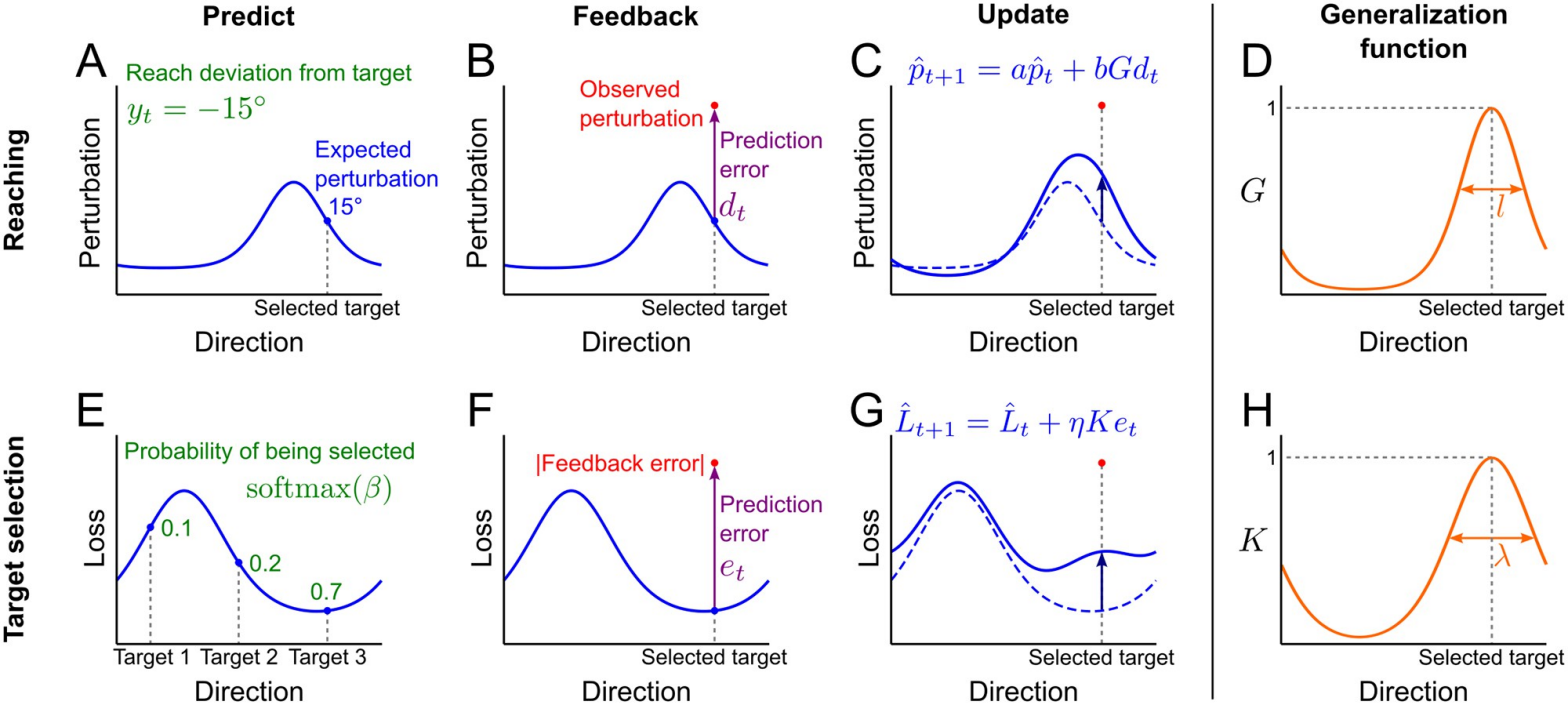
Model of reaching and target selection. Top row: model of reaching. A: On each trial, the model maintains an estimate of the reach perturbation (blue curve) for all target directions. The reach angle counteracts the expected perturbation in the selected target direction (blue dot). B: After movement, the observed perturbation (red dot) is used to calculate the model prediction error (difference between the observed and predicted perturbation, purple). C: The model updates the reach perturbations using the prediction error. The dashed curve shows the previous estimate and the solid curve shows the updated estimate. D: Generalization of the learning is controlled by a kernel function (orange) centered on the selected target direction. Bottom row: model of target selection. E: The model maintains the expected loss (absolute feedback error) as a function of target direction. The probability of selecting each potential target (green numbers) is calculated by applying a softmax function to the expected loss values of the three targets. F: After movement, the observed loss is used to calculate the model prediction error (difference between the observed and predicted loss, purple). G: The model updates the loss function using the prediction error. H: Amount of the loss function update in different directions is controlled by a kernel function (orange) centered on the selected target direction.

The reach model (Fig 5A–5D) assumes that the participants maintain and update, on each trial *t*, an estimate of the reach perturbation ${\hat{p}}_{t}\left(\theta \right)$ for each target direction, *θ* (Fig 5A). Therefore when reaching to a selected target in direction *θ*_*t*_ the estimated perturbation is ${\hat{p}}_{t}\left(\theta_{t}\right)$. We assume that participants choose a reach angle *y*_*t*_ to counteract the expected perturbation:

$$\begin{array}{c} y_{t}=-{\hat{p}}_{t}\left(\theta_{t}\right) \end{array}$$ (1)

Feedback after the movement provides the actual perturbation *p*_*t*_ (assuming participants know their true reach angle, *y*_*t*_) leading to a model prediction error, *d*_*t*_ (Fig 5B):

$$\begin{array}{c} d_{t}=p_{t}-{\hat{p}}_{t}\left(\theta_{t}\right) \end{array}$$ (2)

The model updates the estimates of the perturbation for all directions based on this prediction error (Fig 5C):

$$\begin{array}{c} {\hat{p}}_{t+1}\left(\theta \right)=a{\hat{p}}_{t}\left(\theta \right)+bG\left(\theta ,\theta_{t}\right)d_{t} \end{array}$$ (3)

where *a* is a decay factor and *b* is the learning rate. Crucially, based on previous studies [3], we assume that the amount of learning is highest at the current target direction (*θ*_*t*_) and reduced for directions farther away from *θ*_*t*_. We use a generalization function *G* (Fig 5D) which is a standard periodic kernel function [21] to ensure continuity of the function around the circle. The kernel has a length-scale parameter *l* that controls the fall-off in generalization:

$$\begin{array}{c} G\left(\theta ,\theta^{\prime }\right)=\text{exp}\;(-\frac{2\;{\text{sin}}^{2}\;(\frac{\theta -\theta^{\prime }}{2})}{l^{2}}) \end{array}$$ (4)

At the start of the experiment, the estimated reach perturbation for all target directions was set to 0 (i.e. is well calibrated). We fit the model parameters (*a*, *b* and *l*) by minimizing the mean squared error for each participant given their empirical data. That is, given the series of chosen target directions and perturbations, we calculated the error of the model-predicted reach angle (its difference from the participant’s actual reach angle) in each trial and minimized the mean squared error across all trials.

Reach angle errors derived from the model are plotted as dashed curves in Fig 4A and 4B. Our model qualitatively follows the empirical adaptation for reaching under different bias angles. Note that we did not include a motor noise in Eq (1). Motor noise may make the hand deviate from the intended reach direction but we assume that participants were aware of this at the end of the movement when extracting the perturbation. Importantly, the perturbation depended only on target direction instead of hand direction and therefore the perturbation experienced was independent of the motor noise. So the only effect of motor noise is to potentially add a little noise to the observations of the hand on top of what the model predicts. Since we minimized mean squared error we are allowing for observation noise in the model (the variance left over).

#### Model for target selection

The target selection model (Fig 5E–5H) is based on the policy of choosing the target that would lead to the smallest loss (absolute value of the feedback error). We assume that the participant maintains the expected loss ${\hat{L}}_{t}\left(\theta \right)$ on trial *t* as a function of possible target directions *θ*.

Under the policy of selecting targets with small expected loss, the model determines the probability of selecting each target (*i* = 1, 2, 3) on trial *t* by applying a softmax function to the three expected loss values for the potential target directions on the given trial (Fig 5E):

$$\begin{array}{c} P\left({\text{choice}}_{t}=i\right)=\frac{\text{exp}(-\beta {\hat{L}}_{t}\left(\theta_{ti}\right))}{\sum_{j=1}^{3}\;\text{exp}(-\beta {\hat{L}}_{t}\left(\theta_{tj}\right))} \end{array}$$ (5)

where *θ*_*ti*_ is the direction of the *i*-th potential target on trial *t*. Parameter *β* controls decision noise by setting how much the target choice depends on the expected loss. The model will randomly select targets when *β* = 0; as *β* becomes larger, the target with the smallest expected loss becomes more likely to be selected.

On each trial, the model updates the loss estimate given the outcome of the reach. Let *e*_*t*_ denote the model’s prediction error on trial *t* (Fig 5F):

$$\begin{array}{c} e_{t}=L_{t}-{\hat{L}}_{t}\left(\theta_{t}\right) \end{array}$$ (6)

where *L*_*t*_ is the observed loss (absolute feedback error) on trial *t*.

Given the prediction error, the loss estimate is updated as follows (Fig 5G):

$$\begin{array}{c} {\hat{L}}_{t+1}\left(\theta \right)={\hat{L}}_{t}\left(\theta \right)+\eta K\left(\theta ,\theta_{t}\right)e_{t} \end{array}$$ (7)

where *η* is the learning rate and *K* is again a periodic kernel, now with length-scale parameter λ (Fig 5H):

$$\begin{array}{c} K\left(\theta ,\theta^{\prime }\right)=\text{exp}\;(-\frac{2{\text{sin}}^{2}(\frac{\theta -\theta^{\prime }}{2})}{\lambda^{2}}) \end{array}$$ (8)

The initial loss estimate is set to the observed loss in the first choice trial (trial 1 for Experiment 1 and trial 201 for Experiment 2) equally for all directions.

We fit the model parameters (*η*, λ and *β*) with maximum likelihood for each participant given the empirical choice data. That is we use the participant’s actual target choices and feedback errors in Eqs (6) and (7) to update the loss estimate and maximize the likelihood of the target choices across trials.

**Fig 6:**
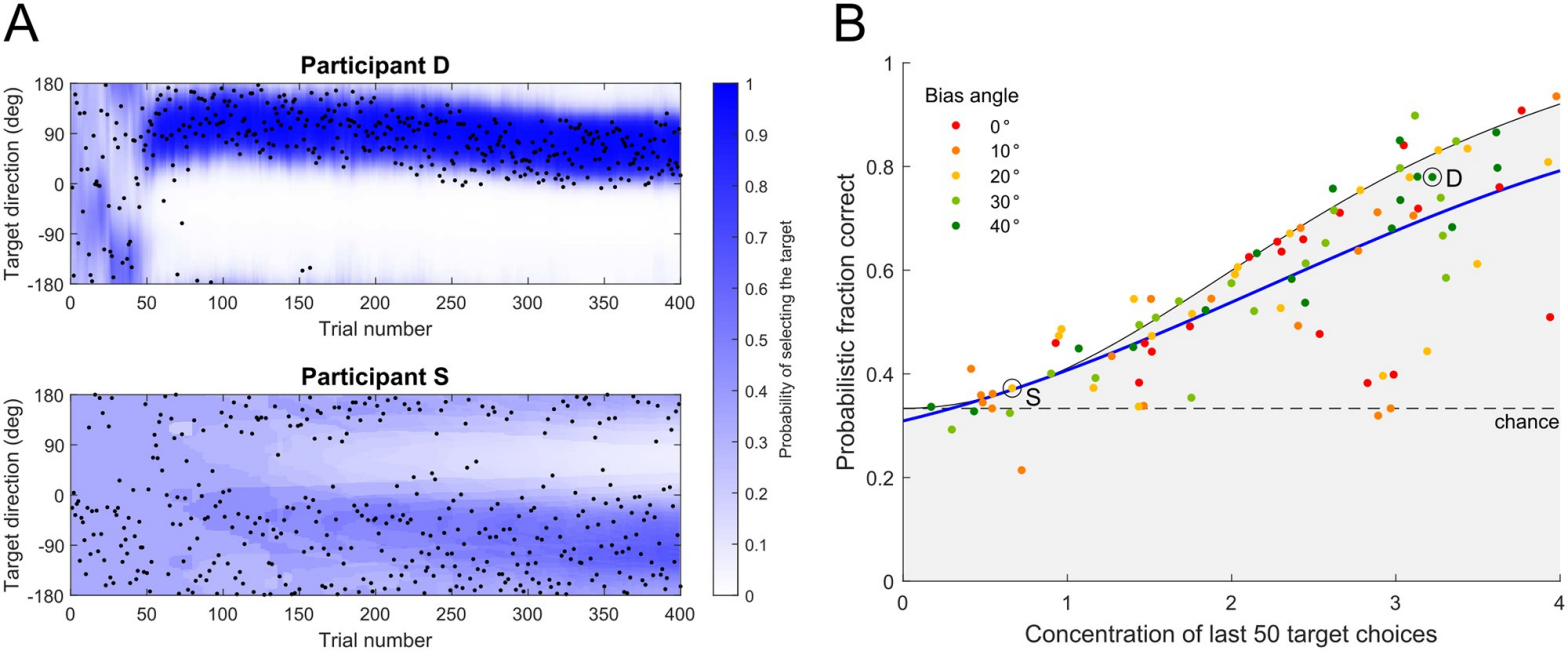
Predictive performance of the target selection model. A: Target choices of two typical participants. Black dots show the selected target direction on each trial. The target choices of participant D gradually became densely concentrated as trial number increased, while the target choices of participant S were relatively scattered until the end of the experiment. The blue gradient shows model-predicted probability of selecting a target if it is located in that specific direction and on that specific trial. The predicted probabilities matched nicely to the actual distribution of target choices for both participants D and S. B: Probabilistic fraction correct of the model predictions on the last 50 target choices of each participant. Boundary of gray shaded area shows the estimated upper bound on the predictive performance as a function of concentration of target choices. Blue curve was fit to all data points (using Gaussian Process Regression) under different bias angles. The mean predictive performance of the model was significantly above chance. The two typical participants D and S (see Panel A) are marked in the figure.

Fig 6A visualizes the model’s outputs for two typical participants. Across trials, the target choices (black dots) of participant D gradually became concentrated around one direction. In contrast, target choices of participant S were more scattered throughout the experiment. The blue shading shows the time-course of the model-predicted probabilities of selecting the targets in different directions (that is for each target direction the probability of choosing that target rather than the two other targets at ±120°). Actual target choices tended to fall within higher probability regions for both participants.

We quantified the predictive performance of our target selection model by the *probabilistic fraction correct* (i.e., the probabilities that the model assigns to the participants’ target choices). Fig 6B shows the probabilistic fraction correct calculated across the last 50 target choices for each participant. The data are plotted as a function of the concentration parameter of a von Mises fit to the last 50 choices for each participant. This concentration parameter determines the upper bound (black curve) on how well any model could perform if it knew the actual distribution of the target choices. The mean performance (blue curve) was above chance (black dashed line at chance = 1/3). Under the notion of minimizing absolute feedback error, our model was able to account for participants’ final target choices across different bias angles.

Model fits are plotted as dashed curves in Fig 4C and 4D. As the model is probabilistic, we derived the plotted values by weighting the angular distance between each optional target and direction *ϕ* by the model-predicted probabilities of selecting each option. Because the empirical data were aligned according to the initial values, we also aligned model fits to these initial values so that the empirical and model data could be directly compared. As shown in Fig 4C and 4D, our model qualitatively follows the target selection behavior under different bias angles.

#### Simulations of the combined model

**Fig 7:**
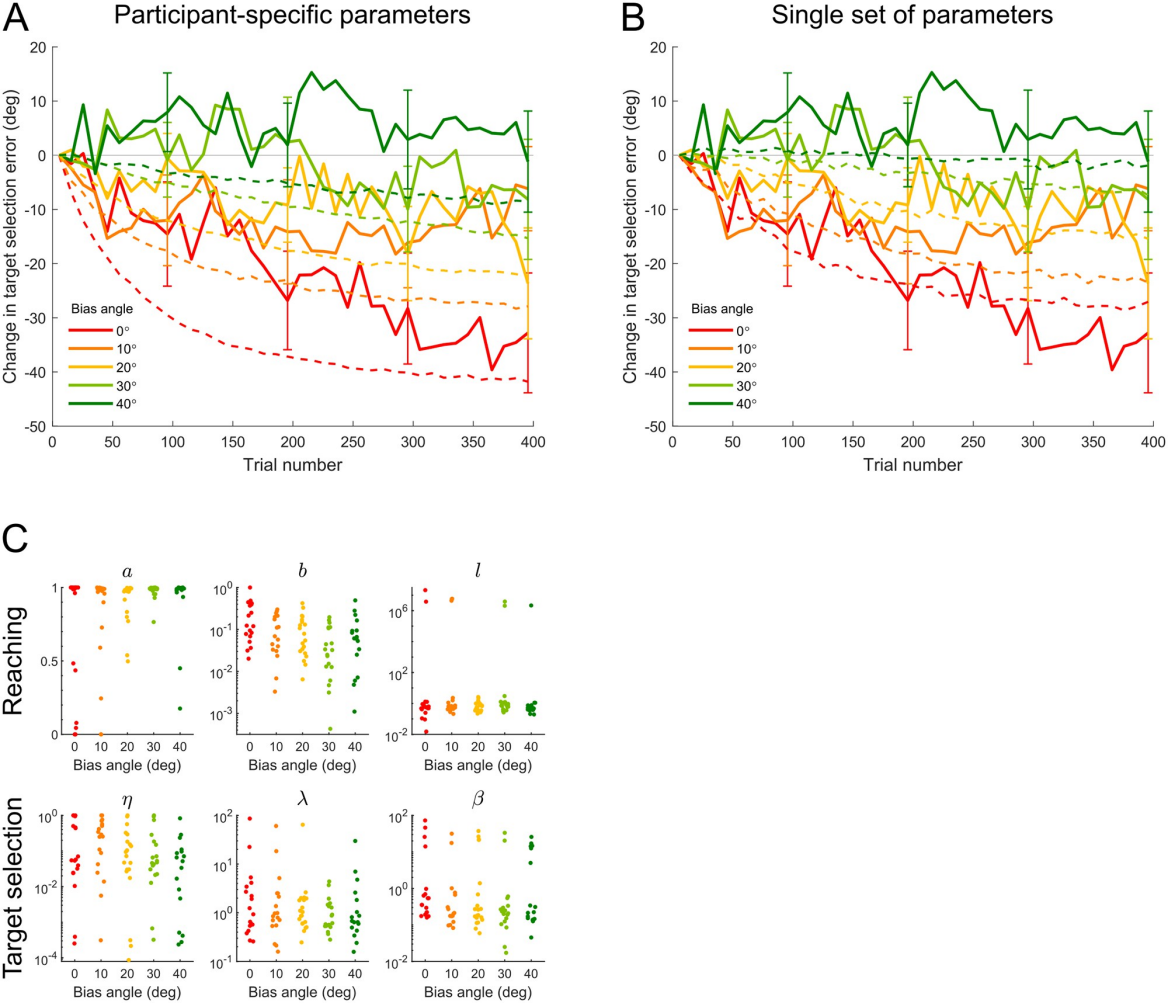
Simulated target selection results across bias angles. A: Change in target selection error as a function of trial number (in consecutive 10-trial bins). Dashed curves show mean across all simulated runs in each bias angle condition (100 runs for each participant using that participant’s best-fit parameters). B: Sample run under a single set of model parameters for all bias angle conditions. Dashed curves show mean across all simulated runs in each bias angle condition (1000 runs under each different bias angle, all using a single set of parameters fit to data pooled across all participants in all conditions in Experiment 1). Solid curves show empirical results (mean ± *SEM* across participants). Both types of simulations qualitatively reproduced the major behavioral result that the learning of the least-noisy direction was inhibited by large bias angles. C: Estimated model parameters for each participant. Upper row shows parameters for the reach model and lower row shows parameters for the target selection model.

The fits of the reach and target selection models shown so far relied on participants’ actual reach and target selection data. That is, for the reach model we used the targets actually selected by each participant, and for the selection model, we used the actual reach errors experienced. To further examine the robustness of the combined reach and target selection models, we ran simulations of the combined model by recursively feeding model-generated target choices and reach angles (instead of participants’ empirical data) back into the model. We first performed model simulation for each participant using their individual best-fit parameters (shown in Fig 7C). Fig 7A shows the simulated data (dashed curves, combining all runs from all participants) as well as participants’ behavioral data (solid curves, also shown in Fig 4C). Although these simulations did not capture the behavior quantitatively as nicely as the model fits shown in Fig 4C, they nevertheless qualitatively reproduced the major behavioral finding that the learning of the least-noisy direction *ϕ* degraded as the bias angle became larger.

To examine whether a *single* set of parameters can explain the differences between groups, we ran sample simulations for all bias angle conditions using the same set of parameters, so that only the bias angle differs in the model simulations across conditions. The parameter values we used were *a* = 0.986, *b* = 0.048, *l* = 0.46, *η* = 0.039, λ = 0.80, and *β* = 0.15, which were the best-fit parameters under data pooled from all 5 groups of participants in Experiment 1. The model simulations (Fig 7B, dashed curves) reproduced the result that large bias angles inhibited the learning of direction *ϕ*. This analysis confirms that a single set of parameters, simulating both target selection and reach behavior, can explain the main features of the data.

#### Alternative models for target selection

The model described above (referred to as Model 1) estimates and minimizes expected absolute feedback error; that is, it assumes that participants focused on the combination of the error in their bias learning and the added noise during target selection. However, it is possible that participants might only focus on learning either the bias or the noise. To explore this issue, we tested two additional models. Model 2 minimizes the remaining bias (i.e. the reach angle error), assuming that participants focused on their reach adaptation (bias learning) performance and ignored the added noise. In contrast, Model 3 minimizes the standard deviation of the perturbations, assuming that participants focused on the noisiness of the perturbation regardless of their reach performance. See Methods for the mathematical details of Model 2 and Model 3. Note that Model 2 and Model 3 may also reproduce some interaction effect between the bias angle and the learning of the least-noisy direction, since the estimation of the bias (mean) and the estimation of the noise (standard deviation) are not computationally independent.

**Fig 8:**
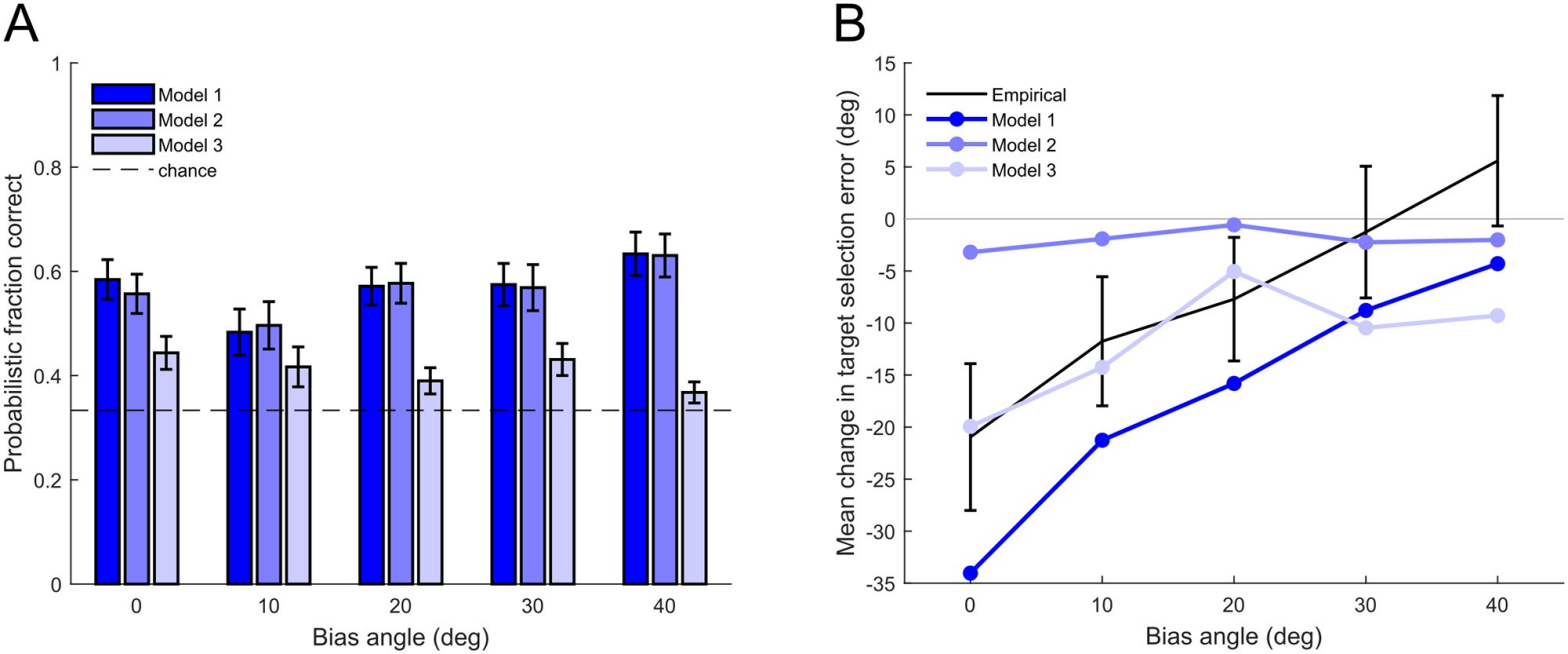
Comparison of different target selection models. A: Average probabilistic fraction correct of model predictions on the last 50 target choices in each bias angle condition and for each model. Dashed line shows value at chance level (1/3). Error bars show *SEM* across participants. B: Mean change in target selection error across the experiment for different bias angle conditions. Black curve shows empirical results (mean ± *SEM* across participants). Blue curves of different brightness show mean across all simulated runs in each bias angle condition (100 runs for each participant) for different target selection models combined with the same reach model.

We first calculated the probabilistic fraction correct across the last 50 target choices for each participant under each model. The overall probabilistic fraction correct across all participants in all conditions were 0.569, 0.566, and 0.410 separately for Models 1, 2, and 3. Fig 8A shows the average probabilistic fraction correct in each bias angle condition and for each model. As illustrated in the figure, Model 1 and Model 2 appear to be comparable in terms of predicting participants’ final target choices, whereas Model 3 performed worse than Models 1 and 2, especially for larger bias angles.

We compared the models’ ability to reproduce the interaction between bias angle and learning of the least-noisy direction. Specifically, we examined the simulated data of each target selection model combined with the same reach model, which are independent of participants’ actual target choices. This is important because repeatedly selecting targets in a particular range of directions will cause a local reduction in reach angle error for those directions. Therefore, if we allow the models to predict participants’ future target choices based on their previous target choices, we cannot evaluate whether Model 2 performed well because participants were actively minimizing reach angle errors when they selected targets or because the model was just reading out participants’ target choice history. Fig 8B shows the mean change in target selection error across different bias angles calculated from simulations under each target selection model. The black curve shows empirical results (also shown in Fig 4D). To compare model performance, we report the goodness-of-fit for each model—a *p*-value smaller than 0.05 means that the simulated data does not fit the empirical trend (see Methods). Out of the 3 models, Model 1 reproduced the empirical trend best (goodness-of-fit: *p* = 0.71). Model 2 produced little learning of the least-noisy direction under any bias angle, neither did it fit the empirical trend well (goodness-of-fit: *p* = 0.0036). Model 3 produced some learning of the least-noisy direction but performed worse than Model 1 in terms of reproducing the trend across bias angles (goodness-of-fit: *p* = 0.029).

Given the model comparison results above, we conclude that during target selection, participants minimized the combination of the remaining bias component and the noise component of the perturbation instead of either component alone.

### Experiment 2: Training with visuomotor rotation bias in all directions

In Experiment 1 we found that very few participants were able to learn the least-noisy direction when the bias angle was 40°. We hypothesized that the reduction in reach angle errors in practiced directions made participants unwilling to explore other directions where adaptation to the bias was poor and hence errors would be large. Therefore, they failed to identify the least-noisy direction. Under these assumptions, if reach angle errors were reduced across all directions by training, it would become easier for participants to learn the least-noisy direction even under large bias angles. Therefore, we designed a second experiment in which we first introduced the bias angle and trained participants in all directions before adding in the noise and target selection trials.

**Fig 9:**
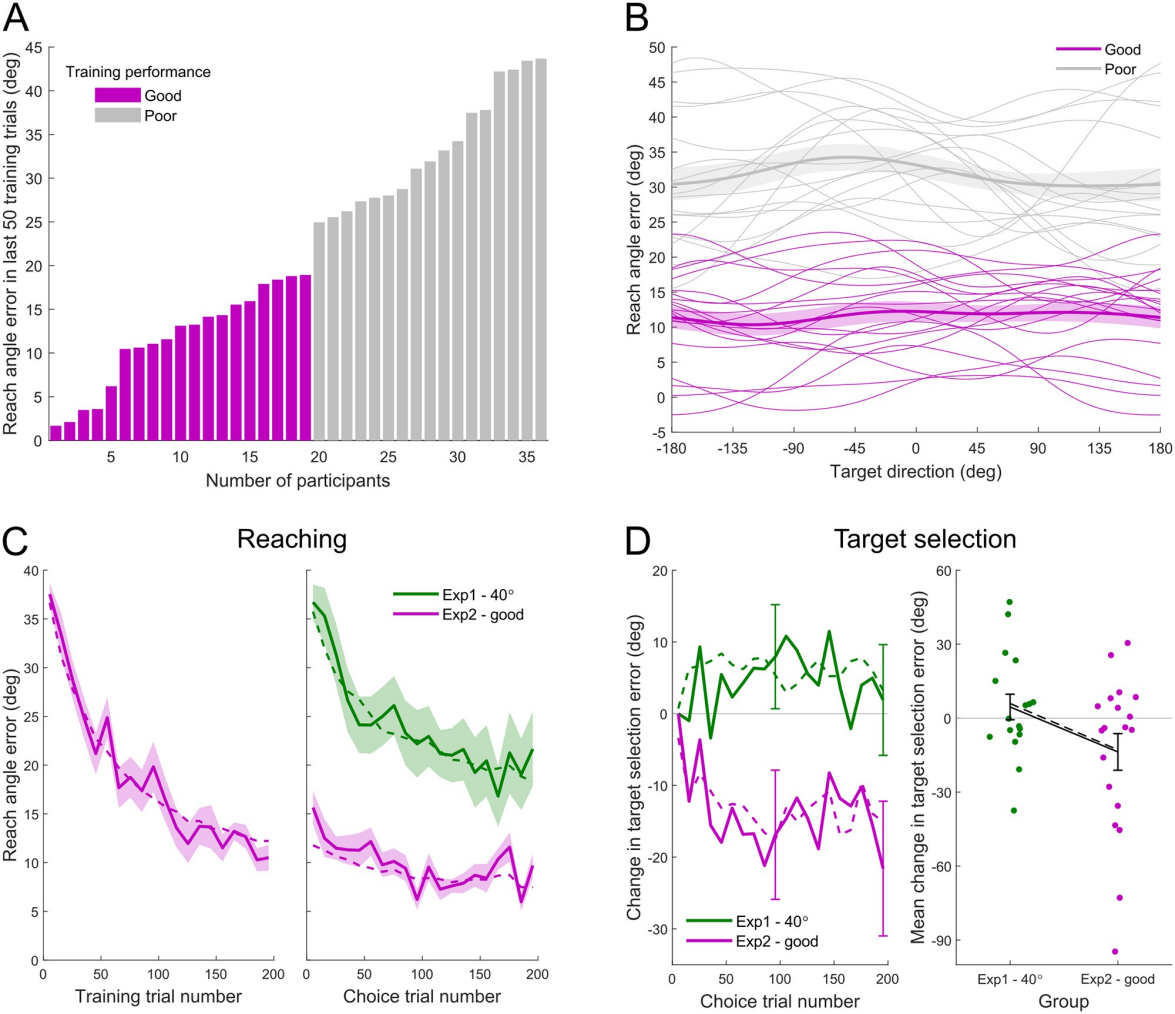
Results from Experiment 2. A: Mean reach angle error in the last 50 training trials for all participants in Experiment 2. Participants with a mean error less than 20° were categorized as good learners (purple filled bars). B: Reach angle error by target direction in the last 50 training trials. Thin curves show data for individual participants. For good learners (purple), reach angle error was reduced to a relatively low level across all directions near the end of the training trials. Curves obtained by applying a periodic kernel smoother. Thick curves show mean for the good learners and the poor learners. Error shadings show *SEM*. C: Reach angle error as a function of trial number (in consecutive 10-trial bins, aligned at the onset of choice trials). Solid curves show empirical results (mean ± *SEM* across participants) and dashed curves show model fits. Good learners (purple) gradually adapted to the bias during training, so that their reach angle errors were smaller than the untrained group (green) during the first 200 choice trials. D: Learning of the least-noisy direction in the good learners (purple) and untrained group (green). Left: Change in target selection error as a function of choice trial number (in consecutive 10-trial bins). Solid curves show empirical results (mean ± *SEM*; for clarity *SEM* only shown at the end of each block) and dashed curves show model fits. Right: Mean change in target selection error across the first 200 choice trials. Colored dots show individual participant’s data. Black solid curve shows mean ± *SEM* across participants. Black dashed curve shows mean of model fits.

Participants were trained for 200 trials with a 40° bias angle (without noise) in random directions before performing 200 choice trials with the same 40° bias but with the addition of target-direction-dependent noise (as in Experiment 1). Fig 9A shows the mean reach angle error in the last 50 training trials (trial 151–200) for each participant in Experiment 2, sorted from smallest to largest. Participants’ performance in the late phase of training varied across a wide range with the mean reach angle error spanning from approximately 0° (full adaptation) to 40° (no adaptation). Out of 36 participants, 19 of them (shown in purple) had a mean reach angle error lower than 20° in the last 50 training trials. We defined these 19 participants as *good learners* and the remaining 17 participants as *poor learners*. Fig 9B shows the reach angle error in different target directions during the last 50 training trials. Each thin curve represents an individual participant. For all good learners (purple), the reach angle error had been reduced to a relatively low level across all target directions near the end of the training trials.

Note that the logic of Experiment 2 requires selecting participants who successfully adapted to the bias angle in all directions in the training trials. That is, the goal was to assess whether, having adapted to the bias, participants could successfully learn to select targets closest to the least-noisy direction, *ϕ*. Therefore, we focused our analyses on the good learners.

Fig 9C shows the reach angle error as a function of trial number for the good learners in Experiment 2 (purple) in the training (left panel) and choice (right panel) trials. The figure includes the 40° group in Experiment 1 (green) in which participants were not pre-trained. As shown in the right panel of Fig 9C, the reach angle error experienced by the good learners after training was lower than that experienced by the untrained participants during the first 200 choice trials, i.e., trial 201–400 in Experiment 2 and trial 1–200 in Experiment 1 (one-tailed two-sample *t*-test on mean reach angle error across the first 200 choice trials for each participant, *p* = 10^−6^).

We then compared the target selection results between good learners after training and untrained participants during the first 200 choice trials. The left panel in Fig 9D shows the change in target selection error (relative to the first 10 choice trials) as a function of choice trial bin, with 10 trials per bin. The right panel in Fig 9D shows the mean change across these 200 choice trials for each participant in each group. Good learners after training were better at learning the least-noisy direction *ϕ* relative to those who were not pre-trained (Fig 9D right, one-tailed two-sample *t*-test, *p* = 0.0286). These results indicate that the poor learning of direction *ϕ* in the 40° group in Experiment 1 is due to large reach angle errors rather than the bias angle per se.

The dashed curves in Fig 9C and 9D show model fits. Our model qualitatively captured the reaching and target selection behavior in Experiment 2.

## Discussion

Our current study aimed to account for participants’ target selection behavior when they perform reaching movements under visuomotor perturbations. In our paradigm, participants perceived an angular displacement between the cursor feedback and the actual reach endpoint in each trial. This perturbation had a fixed bias component and target-direction-dependent noise. Ideally, participants should adjust their reach angle to compensate for the bias and select targets in directions where the noise amplitude was small. This way, participants could reduce the expected absolute feedback error to the lowest possible level. In Experiment 1, we found that participants’ target choices were indeed influenced by the noise amplitude structure. However, as the bias angle grew larger, this influence became weaker. In Experiment 2, we trained participants with 40° bias angle in all directions before letting them freely select targets. After effective training, the influence of noise amplitude structure on participants’ target choices increased relative to the 40° bias angle condition in Experiment 1. Taken together, our results characterize interactions between the separate processes that compensate for errors due to bias or noise, and suggest a relationship whereby errors stemming from the former impact the ability to reduce errors stemming from the latter.

In motor control and learning, noise can reduce the precision of movements and the reliability of feedback signals. Previous studies have reported that humans are able to make optimal motor decisions taking into account the amplitude of both intrinsic and extrinsic noise. For example, motor noise increases with the size of the neural control signal, and humans optimize their eye and arm movement trajectories to minimize endpoint variability in the presence of this signal-dependent noise [10]. Some neurological diseases such as dystonia can increase the level of motor noise, and dystonic people are able to alter their movement strategy accordingly [22]. It has also been shown that people adapt their movement strategies in response to noise added to visual feedback of hand [7–9, 12, 14, 23] or target [11] position. In some studies, the level of added noise varies temporally, and people dynamically adjust their movement strategy according to the current noise level to minimize the negative effect of noise [8, 9, 22, 23]. In other studies, the level of environmental noise varies across a specific dimension of movement options (e.g. spatial direction) and people can learn to select movement options associated with less noise [11–14].

These previous studies on adaptation to non-uniform noise have mostly focused on how people learn to select movement options on the solution manifold, where the mean *signed* movement error is zero. To arrive at the solution manifold, the motor system will typically perform error-based learning, while other learning mechanisms such as reinforcement learning are needed to further reduce mean *unsigned* error along the solution manifold [24]. The main question we aimed to answer in our study is how these two learning processes interact with each other. The bias and noise of the visuomotor perturbation in our paradigm are the two main components of the total movement loss (absolute feedback error angle) in each trial. The bias can be compensated for by altering reach angle relative to the target direction, while the noise can be reduced by reaching in specific target directions. In this way, our paradigm combines error-based motor adaptation and reward/loss-based reinforcement learning of the optimal movement option.

In Experiment 1, with the 0° bias angle condition, we also found that participants learned to reach toward targets near the least-noisy direction during the experiment. When participants encountered a non-zero bias angle, they did not fully adapt to it. Adaptation was best in the preferred target direction and showed limited generalization to other target directions. Even in the preferred direction, group-level mean reach angle errors were above zero at the end of the experiment. These results are consistent with previous findings that adaptation to visuomotor rotation is local [3, 20, 25] and that residual signed errors persist [26–29]. We also found that as the bias angle increased, such that participants started from an initial state further away from the solution manifold, participants were less likely to learn the least-noisy direction. These findings suggest that participants were not able to fully separate the bias and noise components of the visuomotor perturbation—they selected targets that minimized a loss value which is a combination of the remaining error in bias compensation and the noise. We built a computational model which updates the estimated bias angle locally around the selected target direction and updates the loss (expected absolute feedback error) function after each reach. The model assigns larger probabilities of selection for targets with smaller estimated loss values. In accordance with the behavioral results, the model was less likely to select targets in the least-noisy direction as the bias angle grew. We also found that alternative models that minimize the remaining bias or the noise component alone during target selection were not able to account for the behavioral results.

In Experiment 2, participants first experienced a 40° bias angle in all target directions without added noise. Previous work shows that such varied training can benefit the generalization of visuomotor adaptation at the cost of adaptation rate and extent [3, 30]. In our experiment, we found that only a proportion of participants learned to effectively compensate for the bias across all directions such that they approached the solution manifold in all target directions (similar to current manifold 2 in Fig 2A). Relative to the 40° condition in Experiment 1 (similar to current manifold 1 in Fig 2A), these participants were better at learning the least-noisy direction when noise was added after they had reduced the reach angle error in all directions. These results show that learning of the least-noisy direction does not depend on the initial bias (i.e., 40°) but rather the uncompensated bias when noise is introduced.

Our experiments were conducted online with participants making movements with a mouse or trackpad. In common with other online experiments [31, 32], our results match previous findings on the adaptation and generalization of visuomotor rotations performed in a laboratory setting. Using a mouse or trackpad can add anisotropy into movements due to the way computers process the movement data, which could potentially affect target selection. Importantly, we randomized the least-noisy direction for each participant so as to factor out any effects of anisotropy on our results.

Our behavioral and modeling results indicate that participants were able to learn to compensate for the visuomotor bias via error-based learning. At the same time, they selected target directions that minimized the total loss, which was composed of the remaining bias as well as the noise. However, with large bias angles, as the adaptation to the bias gradually reduced the local loss, participants tended to stick to the well-practiced target direction. That is, they were less likely to explore other target directions and learn the least-noisy direction where, in principle, they could get the smallest possible loss. The exploration-exploitation dilemma is typically studied with “multi-armed bandit” tasks such as the Iowa Gambling Task [33], where people need to make decisions under uncertainty and risk. It has been proposed that people can learn a function that maps the options to their expected rewards as well as a decision strategy based on this reward function [34–36]. Our behavioral paradigm and computational models share some common features with these typical tasks including value-based learning, risky decision-making, and exploration-exploitation trade-off. However, our paradigm also involves motor learning, which is distinct from the cognitive tasks in previous studies. In our paradigm, the value associated with each option depends on motor learning performance. That is, to effectively “explore” different target directions, the participant needs to adapt to the visuomotor bias in these target directions. Note that a previous study reported that people can take their anticipated future motor learning performance into account during decision-making to optimize reward [37]. It is conceivable that when participants perceived perturbations with both bias and noise components, they might anticipate that they would be able to fully adapt to the bias given sufficient future practice. In this situation, they might be more willing to search for the target direction where the standard deviation of the visuomotor perturbation is smallest, believing that they could later compensate for the bias, as depicted by one of the alternative target selection models we considered (Model 3). However, this model was not favored under our behavioral data, indicating that participants’ target choices were not solely based on the perceived perturbations—their current motor learning performance was taken into account during the decision-making process.

In our model, we hypothesized that error-based learning, which accounts for the reach behavior, and reinforcement learning, which accounts for the target selection behavior, are carried out simultaneously and interact with each other. Conventionally, these two types of learning are thought to be supported by distinct brain systems, that is, cortico-cerebellar pathways contribute to error-based learning, whereas cortico-striatal pathways contribute to reinforcement learning [38–42]. However, more recent evidence on the neural level suggests that these two processes interact during sensorimotor learning. For example, direct anatomical connections have been found between the cerebellum and striatum [43]. Moreover, studies with rodents have demonstrated that the cerebellum, in addition to processing sensory prediction errors, also encodes reward/loss prediction errors during task performance [44–47]. These findings suggest that error-based learning and reinforcement learning are closely intertwined, which provides an underlying neurological basis for our model.

Our findings provide a new perspective on teaching motor skills in which bias and noise both contribute to the movement loss. If a particular movement is repeatedly practiced, this can reduce the bias for this movement but typically not for other unpracticed movements. Such a practice regime quickly minimizes the loss but it tends to discourage the learner from choosing to explore other movements thereby preventing the learning of bias for movements which may ultimately have less noise. In contrast, if forced to practice a wide range of movements, although this leads to slower reduction in the loss, it can lead to a reduction in bias for all movement options. This can then allow the learner to identify and select the movement that is associated with smaller noise. Which of these two strategies a learner selects may depend on the task horizon (i.e., the number of opportunities the learner will have in the future to perform the motor task). Over a short-term horizon, it may be better for the learner to exploit one movement option, whereas over a long-term horizon it may be better to explore all movement options.

## Methods

### Ethics statement

The research protocols were approved by the Queen’s General Research Ethics Board. Before the experiment, participants gave formal consent by ticking the checkbox “I have read the information above and I am willing to participate.” below the informed consent form displayed on the webpage.

### Participants

We conducted the experiments online on Amazon Mechanical Turk (MTurk). A total of 162 MTurk workers participated in the study. All participants self-reported that they had normal or corrected-to-normal vision and no history of neurological disease. Each participant in Experiment 1 was randomly assigned to one of the five groups; a separate group of participants completed Experiment 2. Participants were paid $3 for completing the experiment, and they were awarded a bonus payment of $0.01 for each successful trial. A total of 34 participants were excluded based on failure to perform the task properly (see details below). The average payment rate was $7 per hour for participants whose data were included in our analysis and $5 per hour for excluded participants.

### Stimuli and procedure

The experimental code was written in JavaScript (p5 and jsPsych). The experiment was conducted in a browser window in full screen mode. Participants were not allowed to use tablets or mobile devices for the experiment. Stimuli were displayed on a 50% gray background. A cross that marked the center of the screen was visible throughout the experiment. Participants used a mouse or trackpad to move a cursor displayed on the screen. We allowed participants to use either a mouse or a trackpad so that they would not be required to use a device they do not normally use. We could introduce a perturbation between the mouse/trackpad position and the displayed cursor. We refer to the mouse/trackpad position (i.e., without the discrepancy) as the “hand” position to distinguish it from the “cursor” position that participants viewed.

#### Experiment 1

In Experiment 1, participants were presented with three targets and chose one as their reach target. During the reach we could add in a visuomotor rotation bias and target-direction-dependent noise on the cursor feedback. Across the five groups we only varied the bias angle applied (0°, 10°, 20°, 30° and 40°).

At the beginning of a trial, participants were asked to move the cursor (white dot) to the central cross and hold it at that position for 500 ms. There were two types of trials: choice trials and probe trials. On *choice trials*, three potential targets then appeared equidistant (35% of the smaller dimension of the screen) from the central cross and equally spaced from each other (120°). The orientation of the three targets was drawn from a uniform distribution on each trial. Note that this design enforced a minimal range of variability (spanning 120°) in the participants’ target choices across trials even if they strictly selected the target closest to a fixed direction in each trial. Each target was circular with a radius of 7.5° (measured from the central cross). Participants were required to choose one of the targets and instructed to move their cursor straight to the target. At the same time as the onset of the targets, the cursor was extinguished and a white circle was continuously displayed whose radius matched the distance of the cursor from the central cross (Fig 1A). When the cursor had moved 50% of the distance from the central cross to the target, the target chosen by the participant was identified as the one closest to the cursor location and the other two targets disappeared. When the white circle made contact with the target, the cursor was displayed for 750 ms. The trial was successful if the cursor overlapped the target. The target was filled with a blue color to indicate success (and turned gray to indicate failure). Participants were encouraged to minimize the distance between the cursor and the target center. After a blank period of 250 ms, the next trial began.

On each trial (*t*) an angular perturbation (*p*_*t*_, relative to the central cross) could be introduced between the hand position and the displayed cursor position. This perturbation could include both a bias (independent of target direction) and noise (target-direction-dependent). Note that we chose to let the noise depend on the selected target direction instead of the reach direction because we wanted the noise to influence target selection. The selected reach angle arises from a secondary decision made once a target is selected. Had we linked the noise to the reach direction, participants would have been faced with a more complex perturbation dependency structure. For each participant we chose a direction *ϕ* (uniformly from the circle) which was the “least-noisy direction”. The noise associated with a given target depended linearly on the angular distance between that target and the least-noisy direction. Thus, if a target happened to be positioned precisely at direction *ϕ*, the target-direction-dependent noise would be zero. Therefore, on each trial, *p*_*t*_ was generated as

$$\begin{array}{c} p_{t}=\alpha +\varepsilon_{t},\;\varepsilon_{t}∼U[-\frac{\delta_{t}}{6},+\frac{\delta_{t}}{6}] \end{array}$$ (9)

where *U* is the uniform distribution, *α* is an offset (bias angle), *ε*_*t*_ is the target-direction-dependent noise, and *δ*_*t*_ is the absolute angular distance from the selected target to direction *ϕ*. Therefore, the range of the uniform noise linearly increased with *δ*_*t*_ (Fig 1C); the maximum possible size of the noise angle was 30° (when *δ*_*t*_ reaches its maximum of 180°). If participants selected targets in directions far from *ϕ*, they would frequently experience relatively large noise angles (much larger than their motor execution noise in reaching) and unlike bias angles, which can be counteracted by visuomotor adaptation, the noise component can only be reduced by selecting targets closer to direction *ϕ*. Participants were not explicitly informed about the existence of the perturbation throughout the experiment.

*Probe trials* were identical to choice trials except only one target was displayed and the location of the target was selected from one of 8 directions equally spaced around the circle. No feedback was provided on probe trials.

Prior to the experiment, participants were given written instructions and performed 5 practice trials (with no perturbation). Real-time instruction captions (e.g., “move the cursor to the cross”, “move the cursor to one of the targets”, …) were displayed during each phase of the practice trials.

After the practice, participants completed 400 choice trials in blocks of 100 trials. They were instructed to take a short break between blocks. The number of trials in which the target was hit and the time left to complete the experiment were displayed after each block (participants had to complete the experiment within 1 hour).

At the end of the 400 choice trials, participants performed an extra block of probe trials designed to measure reaching accuracy across a range of target directions without any further learning. Each of the 8 directions was repeated 3 times in a random order (24 trials).

#### Experiment 2

Whereas in Experiment 1, participants experienced the bias and noise simultaneously (in all choice trials), in Experiment 2 participants were first trained on a 40° bias (in the absence of noise) in all directions before target-direction-dependent noise was added. The general stimuli settings and procedure in Experiment 2 were similar to those in Experiment 1.

There were 200 training trials followed by 200 choice trials, and finally 24 probe trials. In each training trial, there was only one target displayed in a randomly chosen direction. The perturbation between the cursor position and the hand position was a bias angle of *α* = 40° with no noise. The choice trials were the same as choice trials in Experiment 1 with a 40° bias and noise.

### Data analysis

Data were analyzed using MATLAB R2022b. Circular statistics were performed using the Circular Statistics Toolbox [48].

#### Participant exclusion

Some participants repeatedly reached in a single direction regardless of the actual target direction and thus clearly did not attempt to engage in the task. Therefore we excluded participants whose reach direction had a circular standard deviation smaller than 12° in any 20 consecutive trials during the experiment.

For Experiment 2, we further divided the participants into two groups (*good learners* and *poor learners*) based on their mean reach angle error in the last 50 training trials (Fig 9A). The threshold was set to 20°, which divided the participants into approximately two halves. The *poor learners* group was then excluded from further analyses.

Table 1 details the included participants for each group.

**Table 1:** Information of participants included in data analyses.

Group	Experiment 1					Experiment 2	
	0°	10°	20°	30°	40°	All	Good
**Number of participants**	18	18	20	19	17	36	19
**Number of females**	3	9	8	7	4	13	7
**Mean age** (*SD*)	36(11)	34(9)	37(9)	32(8)	38(13)	35(10)	34(9)

#### Trial exclusion

Trials in which the absolute angular difference between the hand endpoint and the selected reach target was larger than 60° were excluded from analysis. Because we identified the selected target based on participants’ hand position *midway* through the movement, it is possible that the final hand position could be closer to a non-selected target. For a choice trial with three targets, this situation could arise from a participant’s change of mind, a slip of the mouse, or by chance if the participant made an ambiguous reach in the middle direction between two targets. For consistency, we also used this criterion in probe and training trials with a single target. The overall percentage of excluded trials for all participants in all experimental conditions was 1.18%. Detailed information about excluded trials is shown in Table 2.

**Table 2:** Percentage of trials excluded from data analyses.

Group	Experiment 1					Experiment 2	
	0°	10°	20°	30°	40°	All	Good
**Choice trials**	0.11%	0.33%	0.40%	0.79%	0.96%	1.75%	1.58%
**Probe trials**	3.01%	3.70%	1.04%	2.19%	1.72%	3.82%	3.29%
**Training trials**						3.35%	2.34%
**All trials**	0.28%	0.52%	0.44%	0.87%	1.00%	2.62%	2.04%

#### Model fitting

We developed models of both reaching and target selection. The details of the models are presented in the *Results* section and we only briefly explain them here.

The model of reaching uses the empirical reach target direction sequence as well as the empirical perturbation sequence as inputs and predicts the hand reach angle for all 424 trials in the experiment. In probe trials, the model continued to decay (with the same decay factor) but was not updated by prediction errors since there was no feedback information. The model has three free parameters: decay factor *a*, learning rate *b*, and generalization length scale *l*. We fit the model by minimizing the mean squared error between model-predicted and empirical hand reach angle sequences (all 424 trials).

The model of target selection uses the empirical target direction sequence as well as the empirical feedback error sequence as inputs and predicts the probability of selecting each of the three target options for all choice trials. The model has three free parameters: learning rate *η*, generalization length scale λ, and softmax parameter *β*. We fit the model by maximizing the log-likelihood of the empirical target choice reach sequence (400 choice trials for Experiment 1 and 200 choice trials for Experiment 2).

All fitting was performed using the fminsearchbnd [49] function (bound constrained optimization) in MATLAB. We report the best-fit parameter set from 50 optimization runs with random initial values.

#### Model simulations

We ran model simulations for the combined reach and target selection model independently of participants’ empirical reach angle and target choices. We first simulated 100 runs of the experiment for each participant using their individual best-fit parameters. Additionally, we simulated 1000 runs of the experiment under each bias angle condition using the same set of parameters (best-fit parameters under data pooled from all 5 groups of participants in Experiment 1). Direction *ϕ* for each simulated run and the target directions in each trial were randomly drawn with the same method in the behavioral experiments.

#### Alternative target selection models

We considered two alternative target selection models (Model 2 and Model 3) other than the one that estimates and minimizes expected absolute feedback error (Model 1, see Results).

Model 2 assumes that participants minimize the expected reach angle error (remaining bias) during target selection. To this end, the model maintains the expected reach angle error ${\hat{R}}_{t}\left(\theta \right)$ on trial *t* as a function of possible target directions *θ*. The model assigns larger probabilities of being selected to targets with smaller (absolute) expected reach angle errors:

$$\begin{array}{c} P\left({\text{choice}}_{t}=i\right)=\frac{\text{exp}(-\beta |{\hat{R}}_{t}\left(\theta_{ti}\right)|)}{\sum_{j=1}^{3}\;\text{exp}(-\beta |{\hat{R}}_{t}\left(\theta_{tj}\right)|)} \end{array}$$ (10)

The expected reach angle error is estimated as the expected *signed* feedback error. After trial *t*, the model updates the estimated expected reach angle error as follows:

$$\begin{array}{c} {\hat{R}}_{t+1}\left(\theta \right)={\hat{R}}_{t}\left(\theta \right)+\eta K\left(\theta ,\theta_{t}\right)\left(R_{t}-\hat{R_{t}}\left(\theta_{t}\right)\right)) \end{array}$$ (11)

where *R*_*t*_ is the observed signed feedback error on trial *t* and *K* is the periodic kernel (see Eq (8)).

Model 3 assumes that participants aim to minimize the standard deviation of the perturbation (noise amplitude), regardless of the reach performance, during target selection. The model uses a one-pass method (the observed data are processed exactly once in order and not stored in the memory after that) to estimate the standard deviation of the perturbation as a function of target direction *θ*. Specifically, the model updates three separate functions of *θ* on trial *t*:

$$\begin{array}{c} \begin{array}{cc} N_{t+1}\left(\theta \right) & =N_{t}\left(\theta \right)+K\left(\theta ,\theta_{t}\right)\\ M_{t+1}\left(\theta \right) & =\frac{N_{t}\left(\theta \right)M_{t}\left(\theta \right)+K\left(\theta ,\theta_{t}\right)p_{t}}{N_{t+1}\left(\theta \right)}\\ S_{t+1}\left(\theta \right) & =\sqrt{\frac{N_{t}\left(\theta \right)S_{t}^{2}\left(\theta \right)+K\left(\theta ,\theta_{t}\right)\left(p_{t}-M_{t+1}\left(\theta_{t}\right)\right)\left(p_{t}-M_{t}\left(\theta_{t}\right)\right)}{N_{t+1}\left(\theta \right)}} \end{array} \end{array}$$ (12)

where *N*_*t*_(*θ*) records the number of samples (trials), *M*_*t*_(*θ*) records the mean of the perturbation, and *S*_*t*_(*θ*) records the standard deviation of the perturbation in target direction *θ* on trial *t*. All three functions are set to 0 for all target directions at the beginning of the experiment. *p*_*t*_ is the observed perturbation on trial *t*, and *K* is again the periodic kernel (see Eq (8)) that controls the range of generalization.

The model assigns larger probabilities of being selected to targets with smaller standard deviations of the perturbation:

$$\begin{array}{c} P\left({\text{choice}}_{t}=i\right)=\frac{\text{exp}(-\beta S_{t}\left(\theta_{ti}\right))}{\sum_{j=1}^{3}\;\text{exp}(-\beta S_{t}\left(\theta_{tj}\right))} \end{array}$$ (13)

We fit Model 2 and Model 3 to each participant’s data and then simulated the experiment by combining each model with the reach model for each participant using the same methods described above for Model 1.

#### Evaluating the target selection model

To quantify the performance of the target selection models we calculated the “probabilistic fraction correct” [50] over the last 50 target choices made by each participant. This is the geometric mean of model-predicted probabilities for the chosen targets, and it is equivalent to the likelihood of the model given the last 50 target choices.

We also estimated an upper bound for any target selection model based on the actual distribution of the target choices for these 50 trials. Specifically, we fit a von Mises (circular normal) distribution to the set of chosen targets. We then used this distribution to calculate the probability of each chosen target. This upper bound therefore takes into account the variability of participants’ choices. If, for example, participants were to choose a different target for two presentations of an identical set of three target options, this would not be possible for any model to accurately predict. Therefore, participants whose von Mises concentration parameter is lower will be fundamentally more difficult to predict by any model and have a low “upper bound” for the probabilistic fraction.

To evaluate whether the model simulations can reproduce the interaction between bias angle and the learning of the least-noisy direction observed in the experiment, we used the following goodness-of-fit measure [51]: We first calculated the signed difference between each participant’s mean change in target selection error (plotted as individual dots in Fig 4D) and the average simulated value across all 100 runs for that participant. We then ran a weighted least-squares (WLS) regression in which the dependent variable was the calculated differences, the independent variable was the bias angle, and the weights were the inverse of the variance. If the simulated values fit well to the trend across different bias angles in the behavioral data, the WLS coefficient for the slope should be 0. We report the *p*-values for the slope coefficient of the WLS regression for each model. If the *p*-value is smaller than 0.05, we reject that the model fits the data.
